# Who’s Driving? Switch of Drivers in Immunotherapy-Treated Progressing Sinonasal Melanoma

**DOI:** 10.3390/cancers13112725

**Published:** 2021-05-31

**Authors:** Sandra N. Freiberger, Patrick Turko, Martin Hüllner, Reinhard Dummer, Grégoire B. Morand, Mitchell P. Levesque, David Holzmann, Niels J. Rupp

**Affiliations:** 1 Department of Pathology and Molecular Pathology, University Hospital Zurich, 8091 Zurich, Switzerland; niels.rupp@usz.ch; 2 Faculty of Medicine, University of Zurich, 8006 Zurich, Switzerland; martin.huellner@usz.ch (M.H.); reinhard.dummer@usz.ch (R.D.); gregoire.morand@usz.ch (G.B.M.); mitchell.levesque@usz.ch (M.P.L.); david.holzmann@usz.ch (D.H.); 3 Department of Dermatology, University Hospital Zurich, 8058 Zurich, Switzerland; patrick.turko@usz.ch; 4 Department of Nuclear Medicine, University Hospital Zurich, 8091 Zurich, Switzerland; 5 Department of Otorhinolaryngology, Head and Neck Surgery, University Hospital Zurich, 8091 Zurich, Switzerland; 6 Department of Otolaryngology, Head and Neck Surgery, Sir Mortimer B. Davis—Jewish General Hospital, McGill University, Montreal, QC H3T1E2, Canada

**Keywords:** mucosal melanoma, sinonasal cancer, oncogenic driver, immunotherapy, tumor heterogeneity, disease monitoring

## Abstract

**Simple Summary:**

Here, we monitored the course of the disease and treatment of sinonasal melanoma patients. Since treatment options are rare, immunotherapy is often the treatment of choice. However, intrinsic or acquired resistance to treatment may occur. We assessed the mutational status of the tumors and metastases during the course of therapy and recognized a switch of the oncogenic drivers to mutant *NRAS* in progressing disease. As a switch of drivers (other than the addition of a second driver) has not been reported yet, longitudinal molecular testing and the awareness of molecular heterogeneity of sinonasal melanoma is crucial.

**Abstract:**

Mucosal melanoma can be driven by various driver mutations in genes such as *NRAS*, *KIT*, or *KRAS*. However, some cases present with only weak drivers, or lacking known oncogenic drivers, suggesting immunotherapy over targeted therapy. While resistance mechanisms to immunotherapy in cutaneous melanoma have been uncovered, including alterations in *JAK1*/*2*, *B2M,* or *STK11*, a switch of oncogenic drivers under immunotherapy has not yet been observed. We report three cases of metastatic sinonasal melanoma that switched oncogenic drivers from *KRAS*, *KIT,* or no driver to *NRAS* during or after immunotherapy, thereby showing progressive disease. One of the cases presented with three spatially separate driver mutations in the primary tumor, whereas the *NRAS* clone persisted under immunotherapy. In comparison, three different control cases receiving radiotherapy only did not show a change of the detectable molecular drivers in their respective recurrences or metastases. In summary, these data provide an important rationale for longitudinal molecular testing, based on evidence for an unforeseen recurrent event of molecular driver switch to *NRAS* in progressing sinonasal melanoma. These findings provide the basis for further studies on a potential causal relation of emerging *NRAS* mutant clones and immunotherapy.

## 1. Introduction

Sinonasal melanoma is a rare melanoma subtype (0.5 cases per Mio/year) [1]. While treatment options for cutaneous melanoma emerged in recent years due to the successful implementation of targeted therapy and immunotherapy (IT) [2], the treatment of metastatic sinonasal melanoma remains challenging. Molecular targets for therapy are infrequent [3], and little is known about responses to immunotherapy [1]. Therefore, metastatic sinonasal melanoma patients are currently treated according to the guidelines for cutaneous melanoma [4]. Besides immunotherapy, targeted therapy with imatinib or nilotinib are options in patients with *KIT* mutations [5,6] or binimetinib for patients harboring *NRAS* mutations [7]. Zaretsky et al. recently reported the acquirement of loss-of-function mutations in *JAK1*/*2* or *B2M*, causing an interruption of the interferon gamma signaling or loss of MHC-I surface expression, respectively, leading to resistance to anti-PD-1 treatment [8]. As resistance mechanisms to targeted therapy with BRAF/MEK inhibitors, mutations in *MAP2K1*/*2*, alternative splice variants of *BRAF*, *BRAF* amplifications or a gain of *NRAS* mutations were detected [9,10]. However, the emergence or selection of *NRAS*-mutant clones in progressing disease has not been shown yet. We report three cases of sinonasal melanoma, initially harboring *KRAS*, *KIT,* or no detectable driver mutation. For the first time, we describe a switch from the initial distinct oncogenic drivers (or no detectable driver) to solely *NRAS* in the course of therapy.

## 2. Materials and Methods

Collection of patient material: The local ethics review board approved this study (BASEC 2020-01663, approved: 30 July 2020) and written informed consent was obtained (BASEC PB_2017-00494, amendment approved: 25 July 2017). Molecular data from the primary tumor of patient 2 and of RT-patient 2 were previously published [3].

Collection of clinical data: All patients were presented regularly at the multidisciplinary tumor board. Clinical workup included molecular analysis to search for driver mutations and possible therapeutic targets. All patients were re-sequenced during the course of metastatic disease.

Molecular analysis: For Next Generation Sequencing, patient material was reviewed by an attending pathologist and the area of interest was marked on a hematoxylin and eosin (H&E) slide. Punch biopsies were then taken from the corresponding FFPE tissue block and DNA was isolated using the Maxwell 16 FFPE Tissue LEV DNA Purification Kit (Promega, Madison, WI, USA). For detection of low frequency mutations with the Oncomine™ Colon cfDNA assay (see below), DNA was isolated from whole sections of the FFPE tumor blocks. DNA was quantified using a fluorometric assay (Qubit, Thermo Fisher Scientific, Waltham, MA, USA). Library preparation was performed using the KAPA HyperPlus Kit (Roche, Basel, Switzerland) according to the manufacturer’s manual. Target capture was performed according to the protocol using a customized probe set (KAPA Hyper Choice, Roche) covering 190 melanoma-typical genes (Table S1) and heterozygous SNPs across the genome to detect copy number changes. HLA-A, HLA-B, and HLA-C loci were covered to conduct HLA typing. The tumor mutational burden (TMB) was calculated using all non-synonymous coding mutations divided by the size of the coding target region of the panel. All libraries were sequenced paired-end (100 bp) on an Illumina NextSeq550 sequencer. Data analysis was performed using a customized pipeline and open-source software [3].

For liquid biopsy, cell-free DNA (cfDNA) was isolated from 2 mL of plasma using the QIAamp circulating nucleic acid kit (Qiagen, Hilden, Germany). The DNA was quantified using a fluorometric assay (Qubit, Thermo Fisher Scientific). NGS on liquid biopsies, as well as NGS on whole sections of the tumors was performed using the Oncomine™ Colon cfDNA Assay (Thermo Fisher Scientific). The libraries were templated and loaded on a 540er chip using the Ion Chef™ instrument (Thermo Fisher Scientific) and sequenced on the Ion S5™ sequencer (Thermo Fisher Scientific). The data were analyzed using the Ion Reporter software version 5.12 (Thermo Fisher Scientific).

Variant reporting focused on main melanoma drivers (MAPK pathway) and potential resistance mutations to approved therapies (e.g., PIK3CA/mTOR pathway).

Histology/Immunohistochemistry: FFPE specimen of 2 µm were stained with hematoxylin and eosin for morphological assessment. Immunohistochemistry was performed using the Bond III automated staining system (Leica), and the monoclonal anti-NRAS (mutated Q61R) antibody SP174 (Abcam, Cambridge, UK). For NRAS staining, pretreatment with H2 was done for 60 min. The recombinant anti-rabbit monoclonal anti-NRAS (mutated Q61R) antibody SP174 (Abcam) was used in a 1:50 dilution. Detection was performed using the Bond Polymer Refine Red Detection Kit (Leica, Wetzlar, Germany). Staining of CD8 and PD-L1 was performed as described previously [3]. The morphological features were evaluated by an experienced head and neck pathologist (NJR). PD-L1 immunohistochemistry was scored as described previously [3]. Numbers of CD8+ T cells were semi-quantitatively assessed (no, low, moderate, high infiltration) in two locations: intratumoral and tumor margin.

FDG-PET/CT: Patients underwent several FDG-PET/CT examinations on different scanners (all manufactured by GE Healthcare, Waukesha, WI, USA).

Dissimilarity and co-ancestry analysis: Dissimilarities were calculated using biallelic SNPs, usually used for CNV analysis, in addition to the 190 MelArray genes. In total, we genotyped 22,246 loci across all chromosomes.

## 3. Results

### 3.1. Patient History Shows Emergence and Selection of an NRAS-Mutant Clone during the Course of Immunotherapy

#### 3.1.1. Case 1

**Figure 1:**
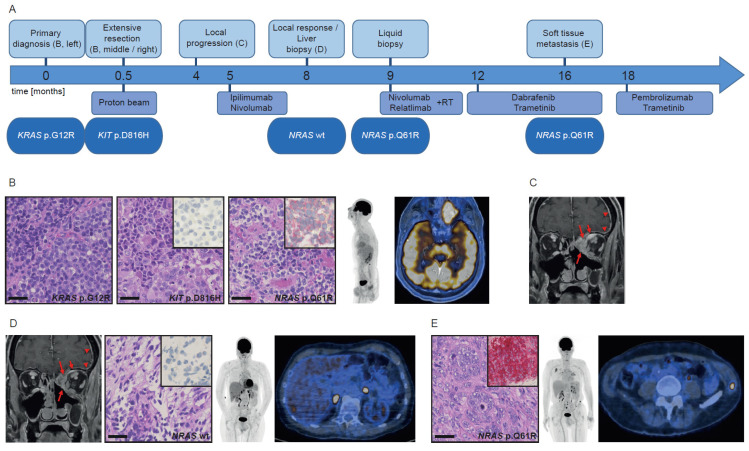
Course of disease, treatment, and morpho-molecular workup of patient 1. (**A**) Timeline. (**B**) Histology of the primary tumor, including NRAS Q61R immunohistochemistry (inserts); left: *KRAS* mutant region, middle: *KIT* mutant region, right: *NRAS* mutant region; PET MIP display and fused PET/CT image at primary diagnosis. (**C**) T1-weighted contrast-enhanced and fat-suppressed MR image at local progression (long arrows: sinonasal tumor mass, short arrows: intraorbital extraconal tumor mass, arrowheads: dural enhancement. (**D**) MR image at local regression (arrows/arrowheads: see above), histology of the liver biopsy, including NRAS Q61R immunohistochemistry (insert); PET MIP display and fused PET/CT image before liver biopsy. (**E**) Histology of the soft tissue metastasis, including NRAS Q61R immunohistochemistry (insert); PET MIP display and fused PET/CT image at progression under kinase inhibitors. Scale bar: 40 µm. wt: wild type.

This 68-year-old male patient (Figure 1A) presented with progressing pressure sensation in the left cheek and orbita. The staging FDG-PET/CT revealed a large, FDG-avid mass in the left ethmoid infiltrating in the cribriform plate and showed absence of regional or distant metastases (Figure 1B). The resection specimen (Figure 1B, left) revealed a *KRAS* p.G12R mutation. Interestingly, the dural part of the tumor (Figure 1B, middle) revealed a *KIT* p.D816H mutation. The multidisciplinary tumor board opted for adjuvant proton beam therapy with 72 Gy. Due to local progression three months later (Figure 1C), immunotherapy with ipilimumab/nivolumab was initiated. Follow-up MRI and FDG-PET/CT after three months showed local tumor response (Figure 1D, left), but liver metastases, which were confirmed by biopsy (Figure 1D, middle and right). One month later, NGS of a liquid biopsy revealed an *NRAS* p.Q61R mutation. Retrospectively, the *NRAS* mutation had not been present in the liver biopsy, which was corroborated by negative staining of the NRAS Q61R antibody (Figure 1D, insert). However, when staining all available blocks from the primary tumor, a minute portion of cells were positive for the NRAS Q61R antibody (Figure 1B, right, insert). Systemic treatment was changed to nivolumab/relatlimab. Subsequent metastases in the bone and lung were additionally treated with stereotactic radiation. Owing to local tumor progression and distant metastases, treatment was changed to dabrafenib/trametinib three months later. An excised soft tissue metastasis of the left flank confirmed the previously detected *NRAS* mutation. Thus, the patient is currently being treated with pembrolizumab/trametinib.

#### 3.1.2. Case 2

**Figure 2:**
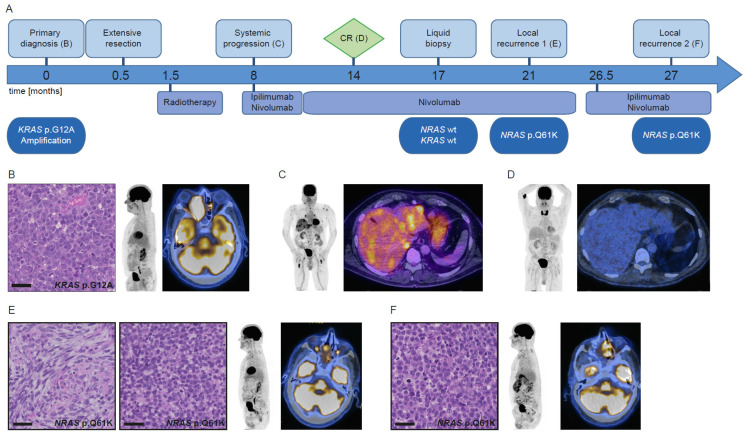
Course of disease, treatment, and morpho-molecular workup of patient 2. (**A**) Timeline. (**B**) Histology of the primary tumor; PET MIP display and fused PET/CT image at primary diagnosis. (**C**) PET MIP display and fused PET/CT image at the start of immunotherapy. (**D**) PET MIP display and fused PET/CT image at CR. (**E**) Histology of the first local recurrence, left: spindle cell shaped morphology, right: monomorphic epithelioid morphology; PET MIP display and fused PET/CT image at first local recurrence. (**F**) Histology of the second local recurrence; PET MIP display and fused PET/CT image at second local recurrence. Scale bar: 40 µm. wt: wild type, CR: complete remission.

This 73-year-old male patient (Figure 2A) presented with watery discharge from the right nostril, pressure on the right eye, and double vision. An MRI scan revealed a mass in the right-sided nasal cavity and maxillary sinus, extending into the orbita and anterior skull base. Biopsy revealed a sinonasal melanoma without regional or distant metastases on FDG-PET/CT (Figure 2B). NGS showed a *KRAS* p.G12A mutation and amplification. Transnasal-transcribriform resection was carried out, followed by intensity-modulated radiation therapy with 70 Gy. On follow-up PET/CT, there was no evidence of local tumor recurrence, but multiple new neck lymph node metastases and distant metastases were found (Figure 2C). A combination of ipilimumab/nivolumab was initiated and after four cycles switched to nivolumab monotherapy (11 cycles). Follow-up PET/CT four months after the start of monotherapy showed complete remission (CR) (Figure 2D). A liquid biopsy taken three months after CR revealed no *KRAS* or *NRAS* mutation. This result can either indicate sustained remission or absence of tumor DNA in the blood for other reasons. More than one year after initiation of immunotherapy, the patient developed a mass in the right nasal cavity, visible both clinically and on PET/CT. No other tumor manifestation was found. Tumor recurrence was confirmed (Figure 2E) and nivolumab treatment was continued. NGS of the biopsy revealed an *NRAS* p.Q61K mutation. Six months later, a subtotal tumor removal was anticipated due to progression. Treatment with trametinib will be started as the *NRAS* mutation is still evident.

#### 3.1.3. Case 3

**Figure 3:**
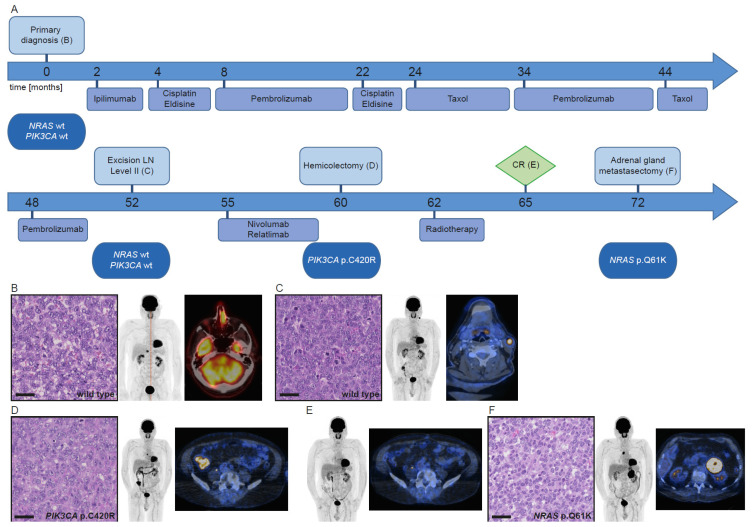
Course of disease, treatment, and morpho-molecular workup of patient 3. (**A**) Timeline. (**B**) Histology of the primary tumor; PET MIP display and fused PET/CT image at primary diagnosis. (**C**) Histology of the lymph node metastasis; PET MIP display and fused PET/CT image at progression after 34 cycles of pembrolizumab. (**D**) Histology of the colon metastasis; PET MIP display and fused PET/CT image at progression under nivolumab/relatlimab. (**E**) PET MIP display and fused PET/CT image at CR. (**F**) Histology of the adrenal gland metastasis; PET MIP display and fused PET/CT image at progression without therapy. Scale bar: 40 µm. wt: wild type, LN: lymph node, CR: complete remission.

This 72-year-old male patient (Figure 3A) underwent surgery of a left-sided endonasal tumor. Histological workup revealed an amelanotic melanoma (Figure 3B), which had no evident driver mutation, but mutations in *JAK2* and *MITF*. For the next two years, the patient underwent various treatment lines that were continuously adjusted depending on disease progression monitored by FDG-PET/CT (Figure 3A).

Forty-eight months after initial diagnosis, a left-sided cervical lymph node metastasis was resected (Figure 3C) and sequenced, which revealed a mutational profile similar to the primary tumor. Due to a progressing metastasis in the cecum, treatment with nivolumab/relatlimab was started. After five months under immunotherapy this lesion was removed surgically (Figure 3D), and molecular analysis revealed a *PIK3CA* p.C420R mutation together with the known *JAK2* and *MITF* mutations. Systemic treatment was discontinued, and another mesenteric lymph node metastasis was irradiated. Thereafter, the patient was considered to be in CR (Figure 3E). Seven months later, a left-sided adrenal gland metastasis was removed surgically (Figure 3F) and confirmed by immunohistochemistry. Molecular analysis revealed an *NRAS* p.Q61K mutation and the known *MITF* mutation, but no *JAK2* mutation. Owing to disease progression, the patient is currently under treatment with ipilimumab/nivolumab.

### 3.2. Mutational Analysis of Different Tumors Reveals Both Intra-Patient Similarities and Molecular Differences

We analyzed heterozygous SNPs across the genome of all tumors, which revealed the relationship of all tumors within each patient. Of note, intra-patient heterogeneity could also be observed (Figure S1).

For each patient we intersected the set of all mutations found in the tumors. Tumors of patient 1 had an overlap of 46.2% (six mutations; Figure S2A). Tumors of patient 2 had an overlap of 33.3% (four mutations; Figure S2B), whereas additional 25.0% (three mutations; Figure S2B) overlapped between the two *NRAS*-mutant tumors. The four tumors of patient 3 had an overlap of 48.1% (13 mutations; Figure S2C). When overlapping the mutations of *NRAS*-mutant tumors from all patients, there were no similarities of the mutational profile between the different patients (Figure S2D). The mutational profile of all tumors is provided in Tables S2–S4. The mutation frequency in synopsis with the tumor cell content revealed the presence of the *NRAS* mutation on one allele, excluding subclonal events.

### 3.3. NRAS Clones either Preexisted or Emerged during the Course of Disease and Therapy

To investigate whether the *NRAS* mutations were already present in the tumor before immunotherapy on a subclonal level, we searched for a low frequency presence of the *NRAS* mutations in the sequencing data from the investigated tissue punches (mean coverage of coding target region: patient 1: 168x (primary diagnosis) and 791x (extensive resection); patient 2: 757x; patient 3: 1371x) but did not find any. Conversely, *NRAS*-mutant tumors did not show any low-frequency traces of the previously detected *KRAS*, *KIT* or *PIK3CA* mutations in the *NRAS* mutant tumors of all patients (mean coverage of coding target region: patient 1: 710x; patient 2: 290x and 1077x; patient 3: 1720x). For the detection of the *NRAS* mutation in the melanoma of patient 1 (*NRAS* p.Q61R), a specific antibody is available. This NRAS Q61R antibody revealed no positivity in the *KRAS*-mutant or *KIT*-mutant part of the primary tumor of patient 1 (Figure S3). However, a minute region was positive in another part, indicative for the *NRAS* mutation. To further investigate this molecular heterogeneity, whole sections of the tumor tissue before immunotherapy were selected and sequenced with a small but highly sensitive NGS panel (Oncomine™ Colon cfDNA Assay) that includes HotSpot regions of *NRAS* and *KRAS* (Table 1).

The tumor of patient 1 revealed the previously detected *KRAS* p.G12R mutation (frequency: 4.6%), confirmed the *NRAS* p.Q61R mutation, previously detected using the corresponding antibody (frequency: 3.3%), and revealed an additional *NRAS* p.G12V mutation (frequency: 7.0%). These findings indicate a molecularly heterogeneous tumor with different subclones (tumor cell content of the whole section: 40%). The previously detected *KIT* mutation is not covered by the target region of the selected panel. The tumor of patient 2 revealed a high proportion of *KRAS* p.G12A mutated cells (64.2%), concordant with our previous results and reflecting an amplification of the mutant allele, but no *NRAS* mutation (tumor cell content of the whole section: 70%). The tumor of patient 3 showed no mutation in *NRAS* (tumor cell content of the whole section: 30%). *MITF* and *JAK2* are not covered by the selected panel.

As all patients underwent additional radiotherapy (RT) during their course of disease, we investigated whether the emergence of the *NRAS* mutation could be an effect of this therapy. We retrospectively identified a control group of sinonasal melanoma patients who underwent solely radiotherapy (*n* = 3). Primary tumors and tumor recurrences or metastases of these three patients before and after radiotherapy were sequenced, whereas no emerging *NRAS* mutation was detected (Table S5).

### 3.4. Tumor Morphology Revealed No Association with Mutational Status or Response to Therapy

Furthermore, we compared the morphology of the different tumors (Figure 1, Figure 2 and Figure 3 histological pictures, Table 2). The initial *KRAS*-mutated melanoma of patient 1 (Figure 1B, left) showed only minor differences to the *KIT*-mutated and *NRAS*-mutated part of the tumor (Figure 1B, middle, right). The *NRAS*-mutated soft tissue metastasis showed a similar morphology, however with obvious chromatin clearing of the nuclei (Figure 1E), while the liver biopsy showed only few vital cells (Figure 1D).

The initial *KRAS*-mutated melanoma of patient 2 (Figure 2B) showed differences in cell size and morphology compared to the first local recurrence, which had a biphasic appearance (Figure 2E), while the second local recurrence (Figure 2F) was similar to the initial tumor.

For patient 3, all manifestations presented a similar morphology (Figure 3B–D), with the *NRAS*-mutant tumor showing smaller cells and lacking the initial prominent nucleoli (Figure 3F).

When comparing the *NRAS*-mutant tumors of the three patients, their morphology did not reveal a distinctive mark to identify this molecular subtype.

### 3.5. CD8+ T Cell Infiltration, PD-L1 Expression and Tumor Mutational Burden (TMB) Do Not Correlate with Therapy Outcome

The tumors before immunotherapy of the responding patients (patients 1 and 2) showed no or only low intratumoral infiltration of CD8+ cells with low to moderate accumulation at the tumor margins, while *NRAS* mutant tumors emerging under therapy showed moderate intratumoral infiltration and moderate to high accumulation of CD8+ cells at the tumor margins. PD-L1 expression was constantly negative or weak in tumors from both patients before and after therapy. The tumor before IT of the intrinsic resistant patient (patient 3) showed a moderate intratumoral infiltration of CD8+ cells with a high number of cells at the tumor margin, while the *NRAS* mutant tumor showed only single intratumoral CD8+ infiltrating cells with moderate presence at the tumor margin. PD-L1 expression was high in both tumor and immune cells before and after therapy (Figure S4). In summary, *NRAS* mutant tumors of the initially responding patients showed a slightly higher CD8+ accumulation in the tumor and at its margins, while the *NRAS* mutant tumor of the intrinsic resistant patient showed a decreased infiltration compared to the WT tumors (Table S6).

Moreover, we calculated tumor mutational burden (TMB) based on our MelArray NGS panel (Table S7). Patients 1 and 2 that initially responded to the treatment had a lower TMB than the intrinsic resistant patient 3. Thus, higher TMB does not seem to correlate with response to IT in our cases.

## 4. Discussion

We have documented the evolution of metastatic sinonasal melanoma in three patients receiving combinational immunotherapy with nivolumab. While two patients (patients 2 and 3) lacked molecular intra-patient heterogeneity before immunotherapy, harboring only a *KRAS* mutation or no evident driver mutation, the primary tumor of patient 1 showed a heterogeneous molecular profile with several different clones and different driver mutations. Both patients 1 and 2 initially responded to immunotherapy, whereas the relapse showed presence of mutant *NRAS*. While the mutation was not detectable in the pre-treatment lesion of patient 2 performing a highly sensitive assay, it seemed to be the dominant, and therefore metastasizing, clone in patient 1. In patient 2, the *NRAS* mutant local recurrence appeared one year after the start of IT with ipilimumab/nivolumab, while the patient was in CR for already 6 months but still under nivolumab treatment. In patient 1 the *NRAS* mutant clone was present in the liquid biopsy four months after treatment initiation and in the later occurring soft tissue metastasis, while the other initially detected drivers were missing. Retrospectively, the *NRAS* mutation was already present on a subclonal level in the primary tumor. Patient 3 had intrinsic resistance to immunotherapy and CR was only reached due to surgery and RT. However, no *NRAS* clone was detectable in the tumor prior to IT, suggesting its emergence after therapy. While the *NRAS* mutation appeared under IT or in close proximity to the end of IT in the first two patients, it occurred late in patient 3 with one year after stop of IT without any further treatment. As this patient had an intrinsic resistance to the IT, it cannot be clarified whether the *NRAS* mutation is directly related to the treatment.

To investigate whether the *NRAS* mutation occurred as a potential effect of radiotherapy (RT), that all patients received at one point during their course of disease, we sequenced tumors before and after RT, but did not find an occurring *NRAS* mutation. Given the rarity of sinonasal melanoma, it is difficult to find a larger group of patients with archived samples before and after a certain treatment. Therefore, our results indicate the occurrence of the *NRAS* mutation rather in the context of immunotherapy than of RT. However, statistical evidence is lacking due to the small cohort size. Nevertheless, we can state that this phenomenon occurred recurrently under real world conditions and is therefore important to know. Further, this finding is very unlikely to occur recurrently in three independent patients of this rare tumor entity just by chance.

Neither clinically nor on FDG-PET/CT did we find lesions suspicious for a second primary tumor that would possibly explain the detection of different oncogenic drivers. While tumor heterogeneity in melanoma is well known, a switch of the driver mutation or a selection for a specific driver mutation in the course of immunotherapy as a possible mechanism of therapy resistance has not been reported yet. It was shown for several cancer types, that intra-patient tumor heterogeneity is limited to passenger mutations, with a consistent main driver in all metastases [11,12,13]. Moreover, when acquiring an additional driver mutation, the initial driver remains present, even within the same tumor cell [14]. Only one study compared primary cutaneous melanoma and corresponding metastases and showed the detection of emerging *NRAS* mutations in two patients. However, no information about treatment of these two patients is available [15]. Due to known intratumoral molecular heterogeneity in melanoma, it can be difficult to assess the mutational status of the whole tumor. NGS results on punch biopsies are potentially biased in tumors with high intratumoral heterogeneity. This was especially evident in patient 1, where we only found a *KRAS* mutation in a punch from the primary resection of the tumor, and afterwards a *KIT* mutation in the extensive resection of the same tumor. Moreover, an *NRAS* mutation became evident in another part of this tumor after using a specific antibody for this mutation. To reduce the bias, whole sections of a tumor or even pooled whole sections of different FFPE blocks of a tumor, instead of punches, can be sequenced. In this way, we were able to detect several clones within the primary tumor of patient 1 (Table 1). Still, it is impossible to sequence every single part of a tumor, especially in a diagnostic setting.

Liquid biopsy is another option to detect different clones. However, liquid biopsy highly depends on the amount of tumor cells in the blood. If this amount is low, the liquid biopsy is likely to remain inconclusive. Moreover, not all clones of a heterogeneous primary tumor may metastasize. Furthermore, it is possible to pick up clones in the blood that originate from a completely different type of cancer (e.g., in a patient with both melanoma and lung cancer). To find an optimal cost-benefit and effort-benefit ratio in the clinical setting, it might be best to do NGS on whole sections of the tumor and disease monitoring using liquid biopsy.

Several reports on the correlation of mutation status and immunotherapy outcome in cutaneous melanoma have been published, but the results remain inconclusive. Kirchberger et al. reported a similar response rate of both *NRAS*-mutant and wild type melanoma to immunotherapy. However, median overall survival was significantly lower in the *NRAS*-mutant group. Moreover, a treatment with MEK inhibitors could lead to a survival benefit of *NRAS*-mutant patients [16]. Shoushtari et al. showed an inferior response of *NRAS* p.Q61 mutant melanoma to anti-PD-1 monotherapy and a trend towards inferior response to a combination of ipilimumab and nivolumab in cutaneous melanoma [17].

In contrast, Johnson et al. found a superior outcome of *NRAS*-mutant patients [18]. Since *KRAS* mutations are rare in melanoma, no results are available for immunotherapy outcome in this group.

The effect of immunotherapy in mucosal melanoma was only investigated in a few reports. However, it seems to be a promising treatment in this type of cancer with very limited therapeutic alternatives. The overall response rate (ORR) to pembrolizumab in mucosal melanoma was shown to be slightly lower than in cutaneous melanoma. However, even CRs were reported [19] similar to our patient 2. When comparing treatment with ipilimumab plus nivolumab in mucosal and cutaneous melanoma, the ORR rate was 37% in mucosal and 60% in cutaneous melanoma, respectively, showing that mucosal melanoma patients may also benefit from this therapy [20]. Several studies reported a correlation of response to immunotherapy and PD-L1 status in various types of cancer [21,22]. Nevertheless, in cutaneous melanoma also patients with negative PD-L1 status can respond to IT, as shown by a previous study with an ORR of 54% and 72% in patients with negative or positive PD-L1 status, respectively [23]. PD-L1 expression in mucosal melanoma is less common than in cutaneous melanoma (44% positivity) as previously demonstrated by Kaunitz et al. [24]. It even seems to be particularly low in the subtype of sinonasal melanoma (20% positivity) [25], which is consistent with our previously published data [3] and the observation in the present study. Still, two of our three patients responded to this treatment.

In all three cases, CD8+ cells were present in the proximity of the tumor before IT and infiltration was shown in resistant tumors. However, a slight increase of CD8+ cells in the initially responding patients was evident in the *NRAS* mutant tumors, while the intrinsic resistant patient showed a slight decrease of CD8+ cells. Whether the CD8+ T cells in the *NRAS* mutant, IT-resistant tumors are exhausted, cannot be concluded from our study. Moreover, with only three patients, it is not possible to draw statistically reliable conclusions about CD8+ cell infiltration and response to IT.

Some associations of activated MAPK signaling and response to IT were recently made, such as reduced T cell infiltration and activity, as shown in breast cancer [26]. Moreover, *NRAS* is upstream of both MAPK and PIK3CA signaling, the latter being involved in reduced CD8+ T cell infiltration and function [27].

Recent studies have shown a correlation of TMB and ORR to immune checkpoint inhibitors. Nevertheless, mucosal melanoma patients, that generally have a lower TMB [28], are able to respond to the treatment [20]. Similar to these findings, the TMB in our two initially responding patients was low, while the intrinsic resistant patient had a slightly higher TMB. Therefore, additional markers to TMB and PD-L1 may play a role in mucosal melanoma.

Resistance to immunotherapy in melanoma is associated with alterations in the interferon gamma pathway or a lack of MHC-I surface expression [8]. We did not detect any alteration affecting the mentioned pathways with our custom NGS panel. While all tumors within one patient shared several passenger mutations, no overlap of passenger mutations among *NRAS*-mutant tumors was evident. Moreover, the morphology of the *NRAS*-mutant tumors differed. This result is consistent with our previously reported lack of morpho-molecular correlation in primary sinonasal melanoma [3].

Our study is limited by the lack of fresh material and patient-derived cell lines for functional testing. Thus, we cannot prove a causal association between the emergence of *NRAS* mutated clones and immunotherapy. In general, activating *NRAS* mutations could be a survival advantage for sinonasal melanoma cells independent of therapy. However, our finding can set the scene for future investigations to clarify the significance of the here detected emerging *NRAS* mutations in the course of immunotherapy.

## 5. Conclusions

Our case series shows the importance of longitudinal testing, preferably by non-invasive liquid biopsy, to monitor not only the presence, but also the change of driver mutations in melanoma patients during the course of disease. Moreover, molecular testing of larger tissue amounts (e.g., whole slide tissue) is preferable over tissue punches in this entity to increase the chances of capturing heterogeneous tumors harboring different relevant driver mutations. Our study also stresses the importance of regular whole-body FDG-PET/CT imaging in the follow-up of sinonasal melanoma patients.

The awareness of the heterogeneity of primary sinonasal melanoma and the fact that a unique driver switch may occur will help to adjust the treatment regimen in time by combining immunotherapy and targeted therapy before the emergence of multiple treatment-resistant metastases. Due to a lack of awareness, the occurrence of this issue can lead to significant discomfiture for pathologists and clinicians in charge and may have dire consequences for patients.

## Figures and Tables

**Table 1:** High sensitivity NGS analysis of whole tumor sections before immunotherapy.

	Mutations	Frequency	Molecular Depth	Tumor Cell Content	Comment
**Patient 1**	*KRAS* p.G12R *NRAS* p.Q61R *NRAS* p.G12V	4.6% 3.27% 7.04%	913x 765x 270x	40%	*KIT* not covered by the assay
**Patient 2**	*KRAS* p.G12A	64.21%	992x	70%	
**Patient 3**	WT			30%	

WT = wild type.

**Table 2:** Comparison of tumor histology.

	Size	Morphology	Nuclei	Other Features	Main Molecular Alteration
**Patient 1**					
Primary diagnosis	medium/large	pleomorphic, atypical	variable size, prominent nucleoli, eccentric nuclei	eosinophilic cytoplasm, abundant mitoses	*KRAS* p.G12R
Extensive resection	medium/large	pleomorphic, atypical	convoluted nuclei, variable size, prominent nucleoli, eccentric nuclei	eosinophilic cytoplasm, abundant mitoses	*KIT* p.D816H
Liver biopsy				few vital cells, myxoid stromal changes	WT
Soft tissue metastasis	medium/large	pleomorphic, atypical	**chromatin clearing of the nuclei**	eosinophilic cytoplasm, abundant mitoses	***NRAS* p.Q61R**
**Patient 2**					
Primary diagnosis	medium/large	monomorphic, atypical	variously distinct nucleoli	eosinophilic cytoplasm	*KRAS* p.G12A/amplification
Local recurrence 1	spindle cells: medium/large; epithelioid cells: **small**	**biphasic appearance** (**spindle cell shaped, monomorphic epithelioid**)			***NRAS* p.Q61K**
Local recurrence 2	small/ medium	monomorphic, atypical			***NRAS* p.Q61K**
**Patient 3**					
Primary diagnosis	medium/large	atypical, epithelioid	prominent nucleoli	eosinophilic cytoplasm, abundant mitoses	WT
LN level II	medium/large	atypical, epithelioid	prominent nucleoli	eosinophilic cytoplasm, abundant mitoses	WT
Hemi- colectomy	medium/large	atypical, epithelioid	prominent nucleoli	eosinophilic cytoplasm, abundant mitoses	***PIK3CA* p.C420R**
Adrenal gland metastasis	**small/** **medium**	atypical, epithelioid	**lacking prominent nucleoli**	eosinophilic cytoplasm, abundant mitoses	***NRAS* p.Q61K**

LN = lymph node, WT = wild type, major differences are printed in bold.

## Data Availability

The data presented in this study are available on request from the corresponding author. The data are not publicly available due to ethical/privacy restrictions.

## Supplementary Materials

The following are available online at https://www.mdpi.com/article/10.3390/cancers13112725/s1, Figure S1: Individual dissimilarity and co-ancestry coefficient of all resected tumors from all patients, Figure S2: Overlap of mutations of the different tumor manifestations within each patient and of the *NRAS* mutant tumors, Figure S3: NRAS Q61R staining of the primary tumor of patient 1, Figure S4: Examples for CD8 and PD-L1 staining, Table S1: List of genes included in the MelArray NGS panel, Table S2: Mutational profile of all tumors of patient 1 acquired by MelArray NGS panel, Table S3: Mutational profile of all tumors of patient 2 acquired by MelArray NGS panel, Table S4: Mutational profile of all tumors of patient 3 acquired by MelArray NGS panel, Table S5: Mutational analysis of sinonasal melanoma before and after immunotherapy, Table S6: Distribution of CD8+ cells and PD-L1 status in all assessed tumors, Table S7: Mutational burden of all samples as calculated by MelArray.
