# Supplementary material for: Who’s Driving? Switch of Drivers in Immunotherapy-Treated Progressing Sinonasal Melanoma
Source: Cancers (Basel). 2021 May 31;13(11):2725. doi: 10.3390/cancers13112725 (PMC8198298; doi:10.3390/cancers13112725)
Supplement: Supplementary file 1 [file cancers-13-02725-s001.zip › cancers-1184033-supplementary.pdf]

Supplementary Appendix

Table of content

Figure S1………………………………………………………………………………………… p.2

Figure S2………………………………………………………………………………………… p.3

Figure S3………………………………………………………………………………………… p.4

Figure S4………………………………………………………………………………………… p.5

Table S1…………………………………………………………………………………………. p.6

Table S2…………………………………………………………………………………………. p.7

Table S3…………………………………………………………………………………………. p.8

Table S4…………………………………………………………………………………………. p.9

Table S5…………………………………………………………………………………………. p.11

Table S6…………………………………………………………………………………………. p.11

Table S7…………………………………………………………………………………………. p.11

Figure S1

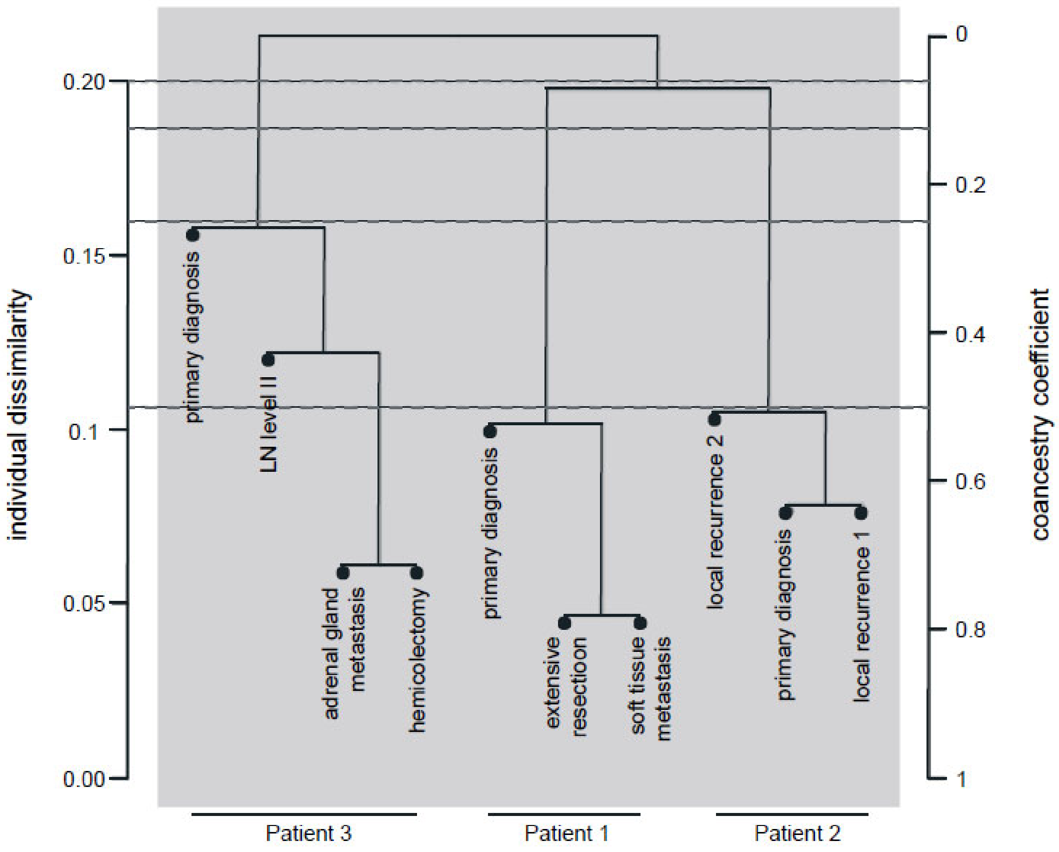

Figure S1: Individual dissimilarity and co-ancestry coefficient of all resected tumors from all patients.

Figure S2

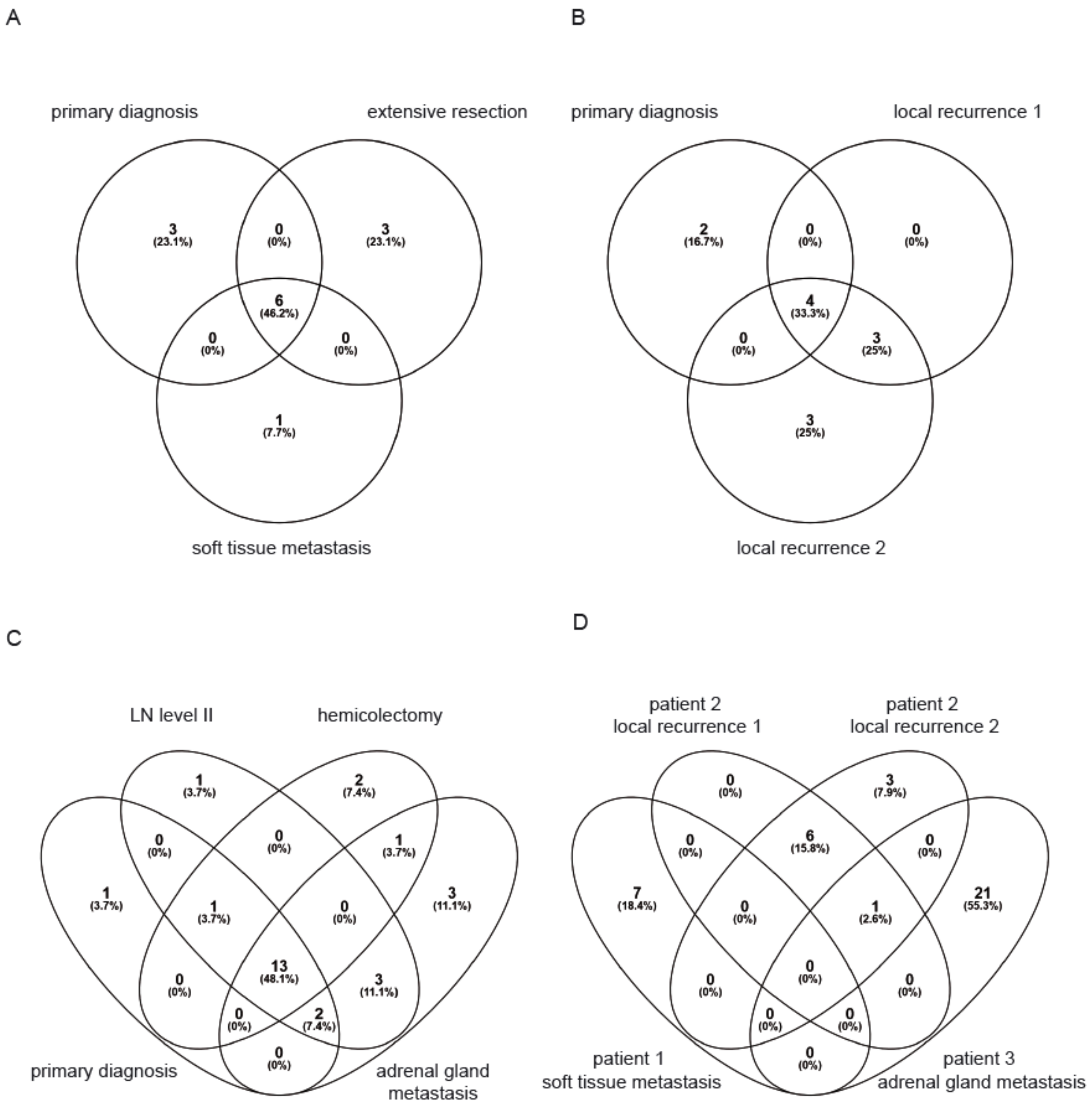

Figure S2: Overlap of mutations of the different tumor manifestations within each patient and of the *NRAS* mutant tumors. A) patient 1, B) patient 2, C) patient 3, D) overlap of mutations of *NRAS* mutant tumors of the three patients. LN: lymph node

Figure S3

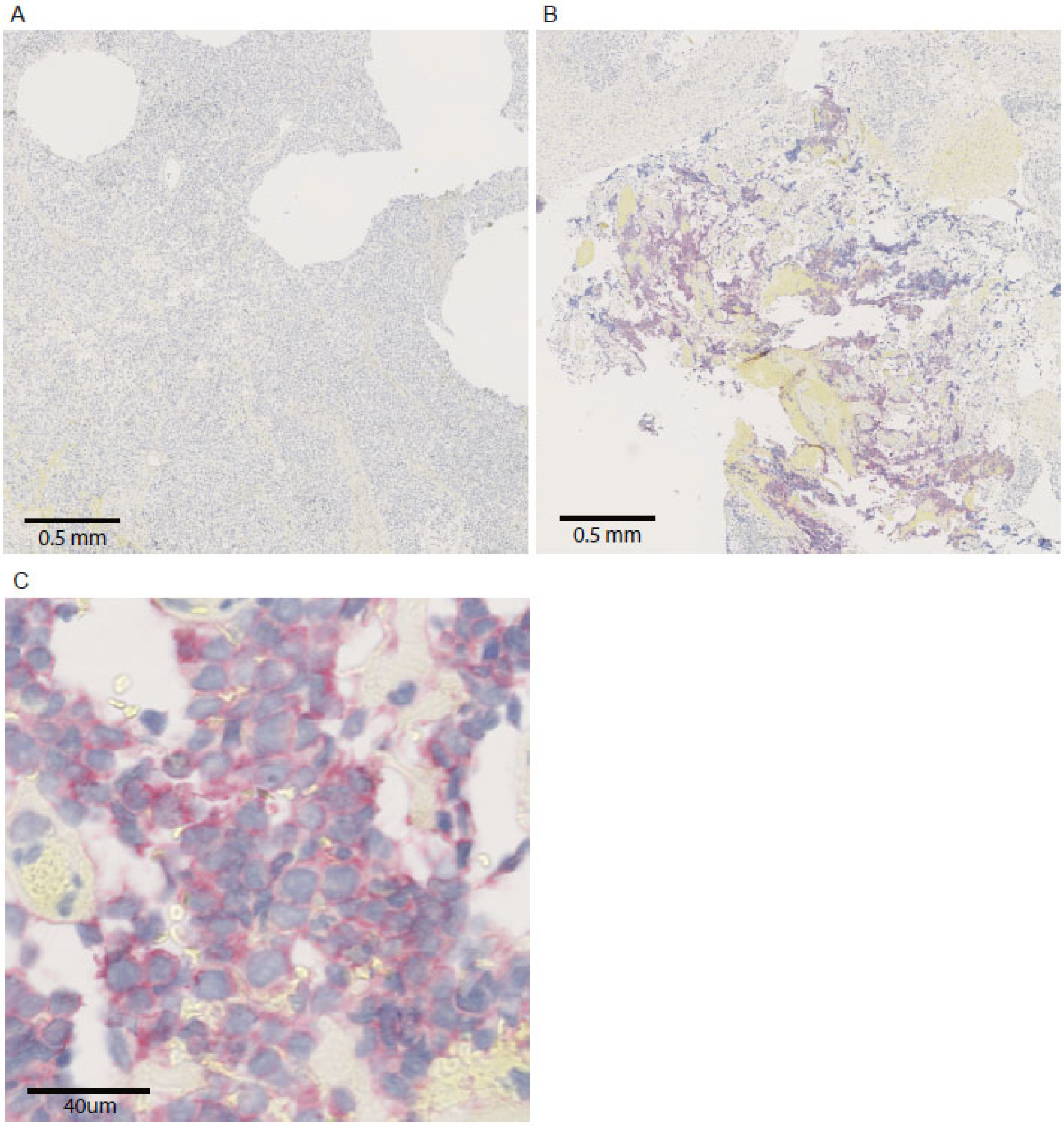

Figure S3: NRAS Q61R staining of the primary tumor of patient 1. A) Overview of the *KIT* mutant region, B) overview of the *NRAS* mutant region, C) higher magnification of the *NRAS* mutant region. Scale bar: 40um

Figure S4

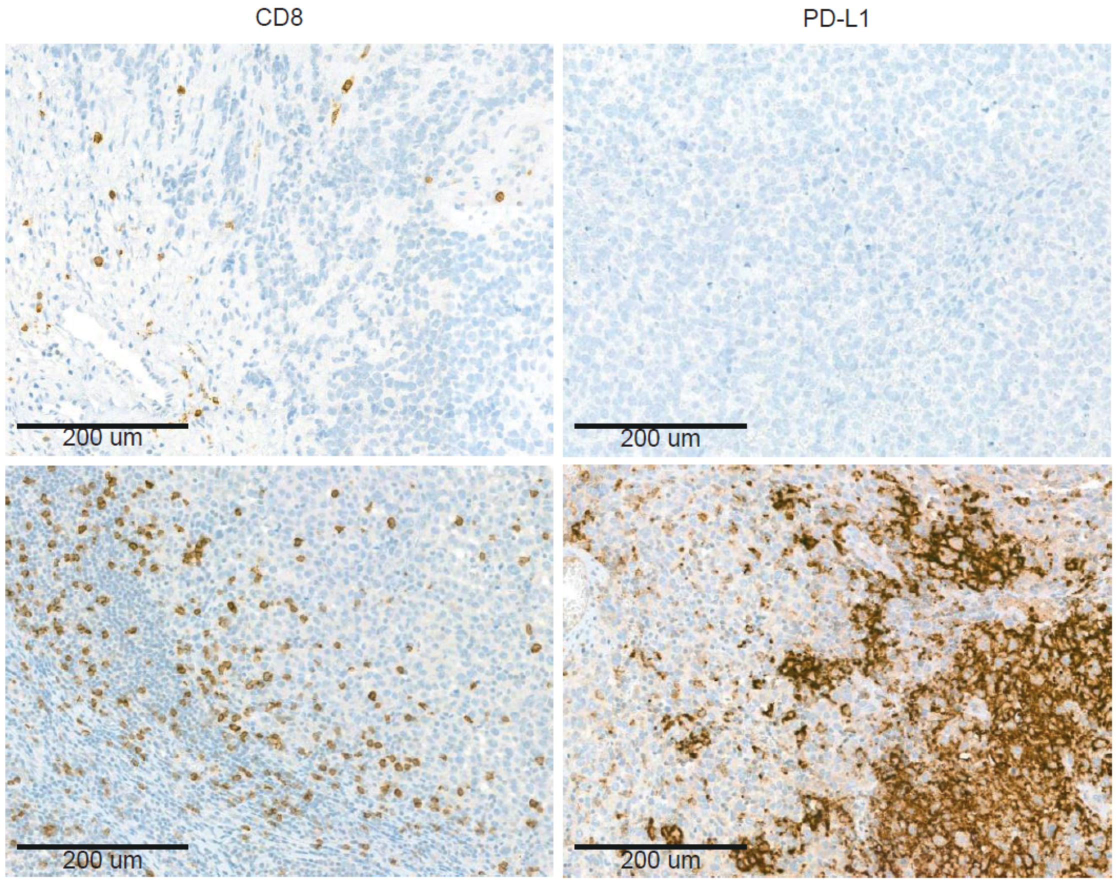

Figure S4: Examples for CD8 and PD-L1 staining. Examples for single CD8+ cells infiltrating within the tumor with moderate accumulation at the tumor margin (upper left), negative PD-L1 staining (upper right), moderate infiltration of CD8+ cells within the tumor and high accumulation at the tumor margin (lower left), and strong PD-L1 staining (lower right).

Table S1: List of genes included in the MelArray NGS panel.

ABCB5	*CCND3*	*ETV6*	*KMT2C*	*PIK3C2A*	*PTPRJ*	*TERF2IP*
ACD	*CDC42*	*EZH2*	*KMT2D*	*PIK3C3*	*PTPRK*	*TERT*
ACVR1C	*CDK4*	*FAM58A*	*KNSTRN*	*PIK3CA*	*RAC1*	*TNRC6B*
AKAP9	*CDK6*	*FANCA*	*KRAS*	*PIK3CB*	*RAD51B*	*TP53*
AKT1	*CDKN1A*	*FBXW7*	*MAP2K1*	*PIK3R1*	*RAF1*	*TRRAP*
AKT2	*CDKN1B*	*FGFR1*	*MAP2K2*	*PIK3R4*	*RASA1*	*TSC1*
AKT3	*CDKN2A*	*FGFR2*	*MAP2K4*	*PIKFYVE*	*RASA2*	*TSC2*
ALK	*CDKN2C*	*FGFR3*	*MAP3K1*	*PKD2*	*RASA3*	*TUSC3*
ANP32C	*CHD8*	*FGFR4*	*MAP3K2*	*PLA2G6*	*RB1*	*TYR*
APC	*CTNNB1*	*FYN*	*MAP3K5*	*PLCB1*	*RET*	*TYRP1*
ARID1A	*CXCL1*	*GNA11*	*MAP3K8*	*PLCE1*	*RICTOR*	
ARID1B	*CYP1B1*	*GNAI2*	*MAP3K9*	*PLEKHG4*	*ROS1*	
ARID2	*CYP7B1*	*GNAQ*	*MAPK1*	*PMEL*	*RQCD1*	
ARID4B	*DCT*	*GNAS*	*MAPK3*	*PMS2*	*SETD2*	
ARID5A	*DCUN1D3*	*HERC2*	*MC1R*	*POLQ*	*SF3B1*	
ASIP	*DDR2*	*HLA-A*	*MET*	*POT1*	*SHOC2*	
ASPM	*DDX3X*	*HLA-B*	*MITF*	*PPARG*	*SLC1A4*	
ATM	*DLG1*	*HLA-C*	*MLH1*	*PPP2R2A*	*SLC45A2*	
AURKA	*DNMT1*	*HRAS*	*MLH3*	*PPP2R2B*	*SMARCA4*	
AURKB	*DNMT3A*	*IDH1*	*MTOR*	*PPP2R5C*	*SMO*	
BAP1	*DNMT3B*	*IGF2R*	*MYCN*	*PPP3CA*	*SOS1*	
BCL2	*DPP3*	*IQGAP1*	*NF1*	*PPP6C*	*SOS2*	
BCL2L12	*DYNC1I1*	*ITGA5*	*NFKBIE*	*PRAME*	*SPRED1*	
BCLAF1	*E2F1*	*JAK1*	*NOTCH2*	*PRKAR1A*	*SPRED2*	
BRAF	*EGFR*	*JAK2*	*NRAS*	*PRKCD*	*SPRY4*	
BRCA1	*EIF1AX*	*JARID2*	*NTRK1*	*PROS1*	*SRC*	
BRCA2	*EIF4A1*	*KDR*	*OCA2*	*PTCH1*	*STK11*	
CBL	*EP300*	*KIT*	*PARP1*	*PTEN*	*TAOK1*	
CCND1	*ERBB2*	*KMT2A*	*PCDHGA1*	*PTPN11*	*TAOK2*	
CCND2	*ERBB3*	*KMT2B*	*PDGFRA*	*PTPRF*	*TERF2*	

Table S2: Mutational profile of all tumors of patient 1 acquired by MelArray NGS panel.

patient 1

**primary diagnosis**		
**gene**	**mutation**	**frequency**
ACVR1C	p.Asn150His	40.1%
ARID5A	p.Pro375Arg	40.5%
ASPM	p.Tyr3353His	50.5%
ASPM	p.Gly1099AlafsTer15	52.8%
KMT2B	p.Met390Thr	40.3%
KMT2C	p.Gly3843Ala	47.4%
KRAS	p.Gly12Arg	78.0%
PKD2	p.Ser804Asn	51.7%
TERT	p.His412Tyr	35.1%
**extensive resection**		
**gene**	**mutation**	**frequency**
ARID5A	p.Pro375Arg	55.4%
ASPM	p.Tyr3353His	64.0%
ASPM	p.Gly1099AlafsTer15	60.8%
IDH1	p.Arg132Cys	48.5%
KIT	p.Asp816His	56.7%
KMT2B	p.Met390Thr	49.9%
NF1	p.Gln1315Ter	42.0%
PKD2	p.Ser804Asn	51.9%
TERT	p.His412Tyr	49.6%
**sof tissue metastasis**		
**gene**	**mutation**	**frequency**
ARID5A	p.Pro375Arg	21.6%
ASPM	p.Tyr3353His	36.2%
ASPM	p.Gly1099AlafsTer15	37.3%
KMT2B	p.Met390Thr	49.8%
NRAS	p.Gln61Arg	91.8%
PKD2	p.Ser804Asn	47.2%
TERT	p.His412Tyr	36.3%

Table S3: Mutational profile of all tumors of patient 2 acquired by MelArray NGS panel.

patient 2

**primary diagnosis**		
**gene**	**mutation**	**frequency**
AKAP9	p.Met3743Ile	63.7%
ALK	p.Glu1400Asp	61.2%
FGFR2	p.Met671Thr	90.0%
KMT2A	p.Arg2191Gln	59.1%
KRAS	p.Gly12Ala	66.7%
PLEKHG4	p.Glu756Gly	35.3%
**local recurrence 1**		
**gene**	**mutation**	**frequency**
AKAP9	p.Met3743Ile	50.8%
ALK	p.Glu1400Asp	51.4%
HERC2	p.Gly4582Glu	86.1%
KMT2A	p.Arg2191Gln	46.4%
NRAS	p.Gln61Lys	47.7%
PLEKHG4	p.Glu756Gly	56.4%
SMARCA4	p.Trp1346Cys	67.8%
**local recurrence 2**		
**gene**	**mutation**	**frequency**
AKAP9	p.Met3743Ile	43.7%
ALK	p.Glu1400Asp	49.4%
ARID1B	p.Ala457GlyfsTer49	23.7%
HERC2	p.Gly4582Glu	29.6%
KMT2A	p.Arg2191Gln	45.8%
NRAS	p.Gln61Lys	16.7%
PLCB1	p.Pro813GlnfsTer5	13.0%
PLEKHG4	p.Glu756Gly	46.2%
PTPRK	p.Arg1039Ser	6.8%
SMARCA4	p.Trp1346Cys	53.1%

Table S4: Mutational profile of all tumors of patient 3 acquired by MelArray NGS panel.

patient 3

primary diagnosis		
**gene**	**mutation**	**frequency**
BRCA2	p.Glu1120Lys	33.1%
GNAS	p.Asp466_Ala467insSerGlyAlaAlaArgAspAlaProAlaAspProAsp	53.5%
JAK2	p.Leu579Phe	30.3%
KMT2A	p.Pro58ArgfsTer92	5.2%
KMT2C	p.Pro3367Ser	15.0%
MAP3K9	p.Ser909Phe	94.0%
MITF	p.Leu400ProfsTer24	74.3%
PARP1	p.His476Tyr	14.0%
PIK3C2A	p.Glu1028Lys	28.1%
PIK3C3	p.Ser128Leu	44.8%
PIKFYVE	p.Glu942Lys	22.9%
PLCE1	p.Asp1102Asn	29.2%
PROS1	p.Arg316Cys	47.4%
PTPRK	p.Glu1373Lys	46.1%
SLC1A4	p.Val289Met	74.1%
SOS1	p.Arg310Cys	23.4%
SOS2	p.Val680Ile	30.1%
**LN Level III**		
**gene**	**mutation**	**frequency**
BCL2	p.Leu185Pro	26.5%
BRCA2	p.Glu1120Lys	18.6%
GNAQ	p.Met1?	6.0%
GNAS	p.Asp466_Ala467insSerGlyAlaAlaArgAspAlaProAlaAspProAsp	55.7%
HERC2	p.Pro339Ser	13.6%
JAK2	p.Leu579Phe	19.1%
KMT2C	p.Pro3367Ser	25.5%
KMT2D	p.Trp5264Cys	23.7%
MAP3K9	p.Ser909Phe	63.1%
MITF	p.Leu400ProfsTer24	72.6%
PARP1	p.His476Tyr	11.8%
PIK3C2A	p.Glu1028Lys	22.0%
PIK3C3	p.Ser128Leu	26.0%
PIKFYVE	p.Glu942Lys	22.2%
PLCE1	p.Asp1102Asn	22.2%
PROS1	p.Arg316Cys	34.6%
PTPRK	p.Glu1373Lys	25.0%
SLC1A4	p.Val289Met	59.1%
SOS1	p.Arg310Cys	23.0%
SOS2	p.Val680Ile	20.6%
**Hemicolectomy**		
**gene**	**mutation**	**frequency**
BRCA2	p.Glu1120Lys	34.7%
JAK2	p.Leu579Phe	30.0%
KMT2C	p.Pro3367Ser	21.1%
MAP3K9	p.Ser909Phe	87.7%
MITF	p.Leu400ProfsTer24	82.7%
PARP1	p.His476Tyr	19.3%

PIK3C2A	p.Glu1028Lys	32.5%
PIK3C3	p.Ser128Leu	37.4%
PIK3CA	p.Cys420Arg	20.2%
PIKFYVE	p.Glu942Lys	23.2%
PLCE1	p.Asp1102Asn	32.9%
PROS1	p.Arg316Cys	43.6%
PTPRJ	c.2153-2A>C	33.5%
PTPRK	p.Glu1373Lys	32.5%
ROS1	c.5249-8C>T	80.5%
SOS1	p.Arg310Cys	26.0%
SOS2	p.Val680Ile	37.2%
**Adrenal gland metastasis**		
**gene**	**mutation**	**frequency**
BCL2	p.Leu185Pro	37.8%
BCLAF1	p.Arg47Cys	6.9%
BCLAF1	c.-10-5_-10-4insCTTC	8.4%
BRCA2	p.Glu1120Lys	22.3%
GNAQ	p.Met1?	5.3%
GNAS	p.Asp466_Ala467insSerGlyAlaAlaArgAspAlaProAlaAspProAsp	33.2%
KMT2C	p.Pro3367Ser	20.1%
KMT2D	p.Trp5264Cys	25.7%
MAP3K9	p.Ser909Phe	83.3%
MITF	p.Leu400ProfsTer24	94.6%
NRAS	p.Gln61Lys	12.3%
PARP1	p.His476Tyr	13.5%
PIK3C2A	p.Glu1028Lys	52.9%
PIK3C3	p.Ser128Leu	35.1%
PIKFYVE	p.Glu942Lys	20.8%
PLCE1	p.Asp1102Asn	25.0%
PROS1	p.Arg316Cys	43.0%
PTPRK	p.Glu1373Lys	42.5%
ROS1	c.5249-8C>T	78.9%
SLC1A4	p.Val289Met	69.4%
SOS1	p.Arg310Cys	20.8%
SOS2	p.Val680Ile	21.9%

Table S5: Mutational analysis of sinonasal melanoma before and after immunotherapy

	Before RT	After RT
**RT-pat. 1**	wild type for *KRAS*, *NRAS*, *KIT*<br>*PTEN* p.V166Sfs*14	wild type for *KRAS*, *NRAS*, *KIT*<br>*PTEN* p.V166Sfs*14, *BRCA2* p.T2125Nfs*4
**RT-pat. 2**	wild type for *KRAS*, *NRAS*, *KIT*<br>*TP53* p.R273C	wild type for *KRAS*, *NRAS*, *KIT*<br>*TP53* p.R273C
**RT-pat. 3**	wild type for *BRAF*, *NRAS*, *KIT*	wild type for *BRAF*, *NRAS*, *KIT*

RT = radio therapy

Table S6: Distribution of CD8+ cells and PD-L1 status in all assessed tumors.

	sample	mutation	CD8	PD-L1
**patient 1**	primary diagnosis	*KRAS* p.G12R	central negative, peripherally moderate	TC0, IC1
	extensive resection	*KIT* p.D916H	central negative, peripherally single positive cells	TC0, IC0
	sof tissue metastasis	*NRAS* p.Q61R	central moderate, peripherally moderate	TC0, IC1
**patient 2**	primary diagnosis	*KRAS* p.G12A	central single pos cells, peripheral modeate	TC0, IC0
	local recurrence 1	*NRAS* p.Q61K	central moderate, peripheral high	TC0, IC1
**patient 3**	primary diagnosis	WT	central single pos cells, peripheral modeate	TC0, IC0
	LN Level III	WT	central moderate, peripheral high	TC2, IC2
	Adrenal gland metastasis	*NRAS* p.Q61K	central single pos cells, peripheral modeate	TC2, IC3

Table S7: Mutational burden of all samples as calculated by MelArray

		number of mutations	mutations / Mb
**patient 1**	primary diagnosis	9	13.8
	extensive resection	9	13.8
	sof tissue metastasis	7	10.8
**patient 2**	primary diagnosis	6	9.2
	local recurrence 1	7	10.8
	local recurrence 2	10	15.4
**patient 3**	primary diagnosis	17	26.2
	LN Level III	20	30.8
	Hemicolectomy	15	23.1
	Adrenal gland metastasis	20	30.8
